# Association of Treadmill Exercise Testing Parameters with PREVENT-Estimated Cardiovascular Risk: A Cross-Sectional Analysis

**DOI:** 10.3390/jcm15062346

**Published:** 2026-03-19

**Authors:** Selen Eşki, Hatice Taşkan, Özkan Eravcı, Şeymagül Karaca, Ahmet Arslan, Erkan Yıldırım

**Affiliations:** 1 Department of Cardiology, Doğubayazıt Dr. Yaşar Eryılmaz State Hospital, Ministry of Health, Ağrı 04402, Türkiye; 2 Department of Cardiology, Haseki Training and Research Hospital, Ministry of Health, Istanbul 34096, Türkiye; 3 Department of Cardiology, Gülhane Training and Research Hospital, Ministry of Health, Ankara 06010, Türkiyeseymagulkaraca@gmail.com (Ş.K.); ahmetarslan2099@gmail.com (A.A.);

**Keywords:** cardiovascular risk assessment, double product, exercise capacity, heart rate recovery, PREVENT equations, primary prevention, treadmill exercise testing

## Abstract

**Background/Objectives:** Atherosclerotic cardiovascular disease (ASCVD) remains the leading cause of death worldwide. The 2023 American Heart Association PREVENT equations represent a contemporary approach to cardiovascular risk estimation, yet they rely on resting clinical and biochemical parameters. This study aimed to evaluate the association between PREVENT-estimated 10-year cardiovascular risk and treadmill exercise testing (TET)-derived physiological variables. **Methods:** We conducted a single-center observational study of 391 participants (mean age 42.9 ± 9.0 years, 56.8% male) who underwent symptom-limited treadmill testing. Ten-year cardiovascular risk was estimated using PREVENT for total cardiovascular disease (CVD), ASCVD, and heart failure (HF). Hierarchical multivariable regression was performed using log-transformed PREVENT risk estimates to quantify the incremental association of exercise capacity (METs), hemodynamic markers (double product), autonomic recovery (heart rate recovery), and the ST/HR index beyond demographic (age, sex, BMI) and extended clinical base models incorporating available PREVENT input covariates. **Results:** Beyond the demographic base model, treadmill parameters were significantly associated with log-transformed PREVENT-CVD risk (ΔR^2^ = 0.026, *p* < 0.001; Cohen’s f^2^ = 0.154). Double product (standardized β = 0.116), HRR at 1 min (standardized β = −0.081), and maximum METs (standardized β = −0.079) were independently associated with risk estimates. However, when the full set of available PREVENT input covariates was included in the base model, the incremental association was negligible (ΔR^2^ = 0.0004, *p* = 0.386), indicating substantial overlap between exercise-derived physiology and PREVENT-embedded clinical information. The incremental association was greatest in participants with intermediate (1–5%) and higher (≥5%) estimated risk (ΔR^2^ = 0.052 and 0.246, respectively). Approximately 14% of participants shifted to a different quartile of estimated risk after inclusion of treadmill data. **Conclusions:** Treadmill-derived physiological parameters are significantly associated with PREVENT-estimated cardiovascular risk, but this association largely reflects shared pathophysiology with PREVENT input variables rather than statistically independent incremental information. Exercise testing may serve as a physiological complement to static risk estimation, particularly in intermediate-risk populations, by providing a dynamic physiological assessment that complements resting clinical measurements. Prospective studies with adjudicated cardiovascular outcomes are needed before clinical implementation.

## 1. Introduction

Despite a steady decline in age-adjusted mortality, atherosclerotic cardiovascular disease (ASCVD) remains the leading cause of death worldwide [1]. Because atherosclerosis develops silently over decades and often becomes clinically evident only at advanced, irreversible stages [2,3], accurate risk stratification during primary prevention is essential for the timely initiation of preventive strategies, particularly in younger and apparently low-risk individuals.

The PREVENT (Predicting Risk of Cardiovascular Disease Events) equations, introduced by the American Heart Association in 2023, represent a major advance in contemporary risk assessment [4,5]. Derived from diverse datasets spanning the past 30 years, PREVENT extends risk estimation to adults as young as 30 years and incorporates critical variables such as renal function and body mass index (BMI), offering a more individualized approach compared to previous models. While its validity has been supported in recent validation cohorts [4,6], these algorithms, like most traditional risk tools, rely primarily on resting clinical and biochemical parameters.

Treadmill exercise testing (TET) provides a unique opportunity to evaluate dynamic physiological responses that are not captured in a resting state. Beyond maximal exercise capacity (METs) and autonomic recovery markers such as heart rate recovery (HRR), TET yields important hemodynamic data, including the double product, a widely used indicator of myocardial workload and hemodynamic stress, and the ST/HR index [7,8,9,10,11]. While exercise testing remains a cornerstone of cardiovascular evaluation due to its accessibility, low cost, and safety [12,13], its incremental association with PREVENT-estimated cardiovascular risk has not yet been systematically examined. Furthermore, it remains unclear whether these physiological parameters exhibit consistent associations across different estimated risk strata and between sexes.

Because PREVENT provides estimated risk probabilities rather than observed clinical outcomes, examining how physiological responses during exercise relate to these calculated risk estimates may offer additional insights into the biological correlates of cardiovascular risk. In this context, treadmill-derived parameters may reflect dynamic cardiovascular responses that complement traditional resting risk factors.

In this study, we aimed to examine the association between PREVENT-estimated 10-year risks (total CVD, ASCVD, and heart failure) and physiological parameters obtained during treadmill testing. Specifically, we sought to determine the extent to which exercise-derived variables explain additional variance beyond the traditional factors incorporated in the PREVENT model. We hypothesized that the integration of dynamic hemodynamic and recovery markers would demonstrate measurable incremental associations with calculated risk estimates, particularly in individuals classified as having intermediate or higher estimated risk by conventional tools.

## 2. Materials and Methods

### 2.1. Study Population and Design

This single-center, observational study was conducted in adult individuals who presented to the outpatient cardiology clinic of Doğubayazıt Dr. Yaşar Eryılmaz State Hospital for cardiovascular screening. The study protocol was approved by the Ethics Committee of Gülhane Training and Research Hospital (Date: 30 September 2025; Decision No: 2025-416) and was conducted in accordance with the Declaration of Helsinki. Written informed consent was obtained from all participants.

The inclusion criteria were:

(1) age ≥ 30 years and
(2) completion of a symptom-limited treadmill exercise test (TET) with a negative result for ischemia.

The exclusion criteria were:

(1) known coronary, peripheral, or cerebrovascular disease;
(2) typical anginal symptoms during testing or positive treadmill findings (ST-segment depression ≥ 1.0 mm in ≥2 contiguous leads);
(3) use of heart-rate-limiting medications (e.g., beta-blockers or non-dihydropyridine calcium channel blockers);
(4) significant arrhythmias or moderate-to-severe valvular heart disease; and
(5) chronic obstructive pulmonary disease requiring oxygen therapy.

By excluding individuals with positive exercise tests, the study cohort was intentionally restricted to clinically stable participants without overt inducible ischemia. This design choice may attenuate associations for ischemia-related parameters such as the ST/HR index. Initially, 391 participants were screened. The primary analysis included 387 individuals with complete data for PREVENT-CVD risk, demographic covariates, and treadmill parameters. HbA1c values were unavailable for 86 participants (22.0%), yielding a reduced sample of 303 for extended models incorporating HbA1c. PREVENT-ASCVD and PREVENT-HF scores were calculable for 388 and 389 participants, respectively. All remaining clinical and exercise variables were complete. Analyses were conducted using available complete cases; no imputation was performed.

### 2.2. Clinical Assessment and Risk Calculation

Demographic characteristics, smoking status, and medical history were recorded at baseline. Physical examination included measurements of weight, height, and resting blood pressure measured in the seated position after at least 5 min of rest using a calibrated sphygmomanometer. Laboratory parameters including lipid profile (total cholesterol, LDL cholesterol, and HDL cholesterol), HbA1c, and creatinine-based estimated glomerular filtration rate (eGFR) were obtained from the hospital’s electronic medical records. eGFR was calculated using the CKD-EPI equation. Ten-year cardiovascular risk was estimated using the 2023 American Heart Association PREVENT (Predicting Risk of Cardiovascular Disease Events) equations for: total cardiovascular disease (CVD), atherosclerotic cardiovascular disease (ASCVD), heart failure (HF).

PREVENT scores were calculated using age, sex, BMI, resting systolic blood pressure, total cholesterol, HDL cholesterol, eGFR, HbA1c (when available), current smoking status, and medication use (antihypertensive and lipid-lowering therapy). Because PREVENT risk is deterministically computed from these input variables, the dependent variable in all regression analyses represents estimated risk rather than adjudicated cardiovascular events. Accordingly, incremental explained variance (ΔR^2^) quantifies the association of treadmill-derived measures with calculated risk estimates, rather than incremental prediction of clinical outcomes.

### 2.3. Treadmill Exercise Protocol and Physiological Parameters

All participants underwent symptom-limited treadmill exercise testing according to the standard Bruce protocol using a commercially available treadmill system (XR450, Cardioline S.p.A., Trento, Italy) with continuous 12-lead electrocardiographic monitoring. A negative test was defined as the absence of typical anginal symptoms and of significant ST-segment changes (i.e., horizontal or downsloping ST-segment depression < 1.0 mm in any lead).

The following treadmill-derived parameters were recorded:

1. Exercise Capacity: Maximum metabolic equivalents (METs), estimated automatically by the treadmill system based on the final workload achieved.
2. Hemodynamic Markers: Maximum heart rate (HR) was defined as the highest heart rate recorded at the end of the exercise stage based on continuous electrocardiographic monitoring. Maximum systolic blood pressure (SBP) was measured by manual cuff sphygmomanometry at peak exercise. Maximal double product was calculated as: peak SBP × peak HR and expressed in ×10^3^ units.
3. Autonomic Recovery: Heart rate recovery (HRR) was calculated as the reduction from peak HR at 30 s, 1 min, and 2 min of recovery. All participants underwent a standardized active recovery protocol consisting of walking at 1.5 mph and 0% incline in the upright position, initiated immediately upon cessation of the exercise phase. Peak HR was defined as the last recorded heart rate at the termination of the exercise stage.
4. ST/HR Index: The ST/HR index was calculated as the ratio of maximal ST-segment depression (μV) to maximum achieved heart rate (beats per minute), yielding units of μV/bpm.

ST-segment depression was measured 60 milliseconds after the J-point (J + 60 ms) across all 12 leads, and the maximum deviation observed in any lead was used for index calculation. In this treadmill-negative cohort, absolute ST-segment depressions were small by definition (<1.0 mm or <100 μV in any single lead). The ST/HR index was therefore evaluated as a continuous marker of subclinical electrocardiographic response rather than a binary ischemia indicator.

### 2.4. Statistical Analysis

Statistical analyses were performed using SPSS version 25.0 (IBM Corp., Armonk, NY, USA) and Python version 3.11 (Python Software Foundation, Wilmington, DE, USA) with the statsmodels library (version 0.14) for regression diagnostics and sensitivity analyses. Statistical significance was defined as a two-tailed *p*-value < 0.05. Normality of PREVENT risk estimates was assessed using the Shapiro–Wilk test, the Jarque–Bera test, and visual inspection of histograms. All PREVENT outcomes exhibited substantial right skewness (PREVENT-CVD skewness = 2.42), with residual non-normality (Shapiro–Wilk *p* < 0.001) and heteroscedasticity (Breusch–Pagan *p* <0.001) in untransformed linear regression models. To address these violations and the bounded nature of risk percentages, natural log-transformation was applied to all PREVENT risk outcomes as the primary analytical approach. Log-transformation achieved approximate residual normality (Shapiro–Wilk *p* = 0.305 for PREVENT-CVD) and substantially reduced skewness (from 1.76 to 0.18). Logit transformation was also evaluated and produced essentially identical results (Supplementary Table S10). Regression diagnostic plots including residual–fitted plots, Q–Q plots, and scale-location plots are provided for both untransformed and log-transformed models in Supplementary Figures S1 and S2. Continuous variables are presented as mean ± standard deviation or median [interquartile range], and categorical variables as frequencies and percentages. Between-group comparisons were performed using Student’s *t*-test or the Mann–Whitney U test for continuous variables and the chi-square test for categorical variables. Associations between treadmill parameters and PREVENT risk estimates were evaluated using Spearman rank correlation coefficients. Partial correlation analyses adjusted for age, sex, and BMI were performed to assess independent relationships. Two sets of hierarchical multivariable linear regression models were constructed to evaluate the association of treadmill-derived parameters with log-transformed PREVENT risk estimates at different levels of covariate adjustment.

#### 2.4.1. Model A (Demographic Base)

Model 0 included age, sex, and BMI.

Model 1 added four treadmill-derived parameters: maximum METs, double product, ST/HR index, HRR at 1 min

This model quantifies the overall association of exercise-derived physiologic measures with PREVENT-estimated risk beyond basic demographic factors.

#### 2.4.2. Model B (Extended Clinical Base)

Model 0 included the full set of available PREVENT input variables: age, sex, BMI, resting systolic blood pressure, total cholesterol, HDL cholesterol, eGFR, current smoking, hypertension, diabetes mellitus.

Model 1 added the same four treadmill parameters.

Because PREVENT scores are deterministically computed from these covariates, a high baseline R^2^ is expected in Model B and should not be interpreted as evidence of superior model performance. The relevant statistic is the incremental ΔR^2^ attributable to treadmill parameters. For each hierarchical model set, incremental explained variance (ΔR^2^) was assessed using the F-change test, with the likelihood ratio test used as a confirmatory measure. Cohen’s f^2^ was calculated to estimate incremental effect size (small < 0.02, medium 0.02–0.15, large > 0.35). Both unstandardized (β) and standardized regression coefficients were reported to allow comparison of relative effect magnitudes across predictors. Multicollinearity was assessed using variance inflation factors (VIF), with VIF > 5 considered indicative of problematic collinearity. Heteroscedasticity-consistent standard errors (HC3) were computed for all models and compared with ordinary least squares estimates to verify the robustness of inference. The incremental association of treadmill parameters with log-transformed PREVENT-CVD risk was evaluated across clinically relevant subgroups defined by: estimated risk level, age, sex, traditional risk factor burden. Risk categories (<1%, 1–5%, ≥5% for PREVENT-CVD) were study-defined strata based on the observed distribution of risk estimates. These thresholds approximate commonly used clinical risk categories but have not been formally validated as PREVENT-specific decision thresholds. Sensitivity analyses using data-driven quartile stratification are presented in Supplementary Table S3. Internal validation was performed using bootstrap resampling (B = 1000) to estimate confidence intervals for ΔR^2^ and regression coefficients. Sensitivity to outcome transformation was evaluated by comparing results across: untransformed linear models (with HC3 robust standard errors), log-transformed models, logit-transformed models. Two-stage residual analyses were conducted by first regressing log-transformed PREVENT risk on the extended clinical covariates and then regressing the resulting residuals on treadmill-derived parameters. This approach provides a complementary assessment of the variance in estimated risk not captured by PREVENT input variables. To evaluate whether the inclusion of treadmill parameters altered the relative ranking of individuals by estimated risk, a distributional shift analysis was performed. Participants were categorized into quartiles based on predicted values from the demographic base model (Model A, Model 0) and the treadmill-augmented model (Model A, Model 1). The proportion of individuals shifting to a different quartile was calculated. Because the dependent variable represents PREVENT-estimated risk rather than adjudicated events, these shifts reflect changes in the ranking of calculated risk estimates and should not be interpreted as formal clinical reclassification in the net reclassification improvement (NRI) sense.

During the preparation of this manuscript, the authors used ChatGPT 5.2 (OpenAI) for language editing. The authors reviewed and edited the output and take full responsibility for the content of this publication.

## 3. Results

### 3.1. Study Population and Baseline Characteristics

A total of 391 participants were screened for eligibility. The primary analysis included 387 individuals with complete data for PREVENT-CVD risk estimates, demographic covariates, and treadmill-derived parameters. HbA1c values were unavailable for 86 participants (22.0%), resulting in a reduced sample size (*n* = 303) for extended models incorporating HbA1c. PREVENT-ASCVD and PREVENT-HF scores were available for 388 and 389 participants, respectively. All other clinical and exercise variables were complete. Analyses were conducted using complete-case datasets without imputation. Baseline demographic, biochemical, exercise-related, and PREVENT risk characteristics of the study population are summarized in Table 1.

The mean age of the study population was 42.9 ± 9.0 years, and 222 participants (56.8%) were male. The mean body mass index (BMI) was 27.3 ± 3.5 kg/m^2^, indicating that most individuals were overweight. Current smoking was reported by 36.6% of participants, while the prevalence of hypertension, diabetes mellitus, and hyperlipidemia was relatively low (7.9%, 3.1%, and 2.0%, respectively). Biochemical parameters reflected a generally favorable metabolic profile, with mean total cholesterol of 186 ± 35 mg/dL, LDL cholesterol of 104 ± 32 mg/dL, preserved renal function (mean eGFR: 103.8 ± 12.6 mL/min/1.73 m^2^), and a median HbA1c of 5.60% [5.40–5.90].

With regard to exercise testing, participants demonstrated relatively high functional capacity, with a median maximum METs value of 12.0 [9.4–14.8]. The mean peak heart rate was 161 ± 15.1 bpm, and median peak systolic blood pressure was 150.0 [139.0–164.0] mmHg. Median heart rate recovery (HRR) values were 8.0 bpm at 30 s, 28.0 bpm at 1 min, and 50.0 bpm at 2 min. The mean double product was 20.0 ± 3.0 × 10^3^, and the median ST/HR index was 0.70 [0.30–1.10]. Baseline PREVENT risk estimates were low overall, with median PREVENT-CVD, PREVENT-ASCVD, and PREVENT-HF scores of 1.8%, 1.2%, and 0.6%, respectively, reflecting a predominantly primary prevention cohort.

Because PREVENT risk estimates were markedly right-skewed (skewness = 2.42), regression analyses were conducted using log-transformed outcomes as specified in the Methods section. Diagnostic evaluation confirmed that log transformation achieved approximate residual normality (Shapiro–Wilk *p* = 0.305) and reduced heteroscedasticity (Supplementary Figures S1 and S2). All regression results reported hereafter are therefore based on log-transformed outcomes unless otherwise stated.

### 3.2. Hierarchical Multivariable Regression Analysis: Association of Treadmill Parameters with PREVENT-Estimated Risk

Primary Analysis (Model A: Demographic Base): The association of treadmill-derived parameters with log-transformed PREVENT-CVD risk was evaluated using hierarchical multivariable linear regression (Table 2). In Model 0, age, sex, and BMI were entered as base covariates. In Model 1, maximum METs, double product, ST/HR index, and HRR at 1 min were added.

Because the dependent variable is PREVENT-estimated risk rather than observed cardiovascular events, these analyses quantify the incremental association of treadmill-derived physiologic measures with calculated risk estimates. In the demographic base model (Model 0), age (β = 0.089, *p* < 0.001) and male sex (β = 0.602, *p* < 0.001) were strongly associated with higher log-transformed PREVENT-CVD risk. This base model explained 80.5% of the variance. The high baseline R^2^ is expected when modeling the output of a deterministic risk equation, as PREVENT scores are largely driven by age and sex. Accordingly, ΔR^2^ represents the more informative statistic in this analysis.

After addition of treadmill parameters (Model 1), three exercise-derived variables were independently associated with PREVENT-CVD risk estimates: maximum METs (β = −0.036, *p* = 0.002; standardized β = −0.079), double product (β = 0.038, *p* < 0.001; standardized β = 0.116), and HRR at 1 min (β = −0.007, *p* < 0.001; standardized β = −0.081). The ST/HR index did not reach statistical significance (β = 0.019, *p* = 0.501). Sequential hierarchical models evaluating the incremental contribution of individual exercise parameters are presented in Supplementary Table S2.

Based on standardized coefficients, double product showed the largest incremental contribution among treadmill variables, followed by HRR at 1 min and maximum METs. The addition of treadmill parameters resulted in a statistically significant increase in explained variance (ΔR^2^ = 0.026; F-change = 14.58; *p* < 0.001), corresponding to a medium effect size (Cohen’s f^2^ = 0.154). No meaningful multicollinearity was observed among predictors (all VIF < 2). Heteroscedasticity-consistent (HC3) standard errors confirmed that all significant associations remained robust (Supplementary Table S10).

#### Extended Clinical Adjustment (Model B: Full PREVENT Covariates)

To test whether treadmill-derived parameters are associated with PREVENT-estimated risk beyond the clinical variables already embedded in the PREVENT algorithm, extended models were constructed with the full set of available PREVENT input covariates as the base (Table 3).

When the full set of available PREVENT input covariates was used as the base model, the baseline R^2^ reached 0.966, reflecting the near-deterministic relationship between the PREVENT algorithm inputs and its computed output. In this context, no treadmill-derived parameter retained statistical significance, and the incremental variance explained was negligible (ΔR^2^ = 0.0004; F-change = 1.04; *p* = 0.386). The likelihood ratio test confirmed no significant improvement in model fit (χ^2^ = 4.30, *p* = 0.366). Consistent patterns were observed for PREVENT-ASCVD (ΔR^2^ = 0.0003, *p* = 0.474) and PREVENT-HF (ΔR^2^ = 0.0006, *p* = 0.379).

This finding indicates that the physiological information captured by treadmill parameters substantially overlaps with the clinical variables already incorporated in the PREVENT equations, particularly resting blood pressure, metabolic markers, and smoking status.

### 3.3. Correlation Analyses Between Exercise Parameters and PREVENT-CVD Risk

Bivariate and adjusted correlations between treadmill-derived parameters and PREVENT-CVD risk are presented in Supplementary Table S1.

In unadjusted analyses, HRR at 1 and 2 min, double product, and maximum METs showed significant associations with PREVENT-CVD scores. After adjustment for age, sex, and BMI, maximum METs (r = −0.220, *p* < 0.001), ST/HR index (r = 0.185, *p* < 0.001), and double product (r = 0.278, *p* < 0.001) remained significantly correlated with risk estimates, whereas the association of HRR at 1 min was no longer statistically significant.

### 3.4. Subgroup Analyses

#### Clinical Risk Categories and Risk Factor Burden

The incremental association of treadmill parameters with log-transformed PREVENT-CVD risk across clinically relevant subgroups is shown in Table 4.

In participants with low estimated risk (<1%), treadmill parameters were not significantly associated with additional variance in PREVENT-CVD estimates (ΔR^2^ = 0.009, *p* = 0.794). In contrast, participants with intermediate (1–5%) and higher (≥5%) estimated risk demonstrated statistically significant incremental associations (ΔR^2^ = 0.052 and 0.246, respectively; both *p* < 0.001). Among individuals without traditional risk factors, treadmill parameters explained an additional 2.3% of variance, whereas in those with at least one risk factor the incremental contribution increased to 5.0% (both *p* < 0.001).

### 3.5. Age and Sex Stratification

Participants aged ≥45 years exhibited a greater incremental association compared with younger individuals (ΔR^2^ = 0.113 vs. 0.031; both *p* < 0.001). The association of treadmill parameters with PREVENT-CVD risk was significant in both sexes, with a numerically greater incremental contribution observed in women (ΔR^2^ = 0.038) than in men (ΔR^2^ = 0.022) in the log-transformed model.

### 3.6. Clinical and Risk Profiles Across Subgroups

Detailed comparisons of exercise parameters and PREVENT risk scores across clinical subgroups are presented in Supplementary Table S4A,B.

Women exhibited faster HRR at 1 min than men (*p* = 0.002). Smokers and individuals with hypertension or diabetes demonstrated impaired HRR and reduced exercise capacity. Maximum METs was significantly lower in participants with diabetes and dyslipidemia. Double product was higher in men and smokers.

PREVENT scores were consistently higher in men and smokers. However, participants with diagnosed cardiometabolic conditions such as hypertension and diabetes exhibited lower estimated risk which may reflect the effects of ongoing medical treatment.

### 3.7. Associations with Traditional Risk Factors

Spearman correlation analyses are presented in Supplementary Table S5. Maximum METs was inversely correlated with age, hypertension, diabetes, and BMI. HRR parameters were negatively correlated with age and cardiometabolic risk factors, whereas double product showed positive associations with age, sex, and BMI.

### 3.8. Robustness, Sensitivity, and Distributional Analyses

#### 3.8.1. Internal Validation

Internal validation using bootstrap resampling (B = 1000) confirmed stable incremental contributions for the demographic base model (bootstrap ΔR^2^ 95% CI: 0.014–0.045; Supplementary Table S6A). Bootstrap confidence intervals for individual treadmill coefficients are provided in Supplementary Table S6B.

#### 3.8.2. Transformation Sensitivity

To assess the robustness of results across analytical approaches, untransformed linear models with heteroscedasticity-consistent (HC3) standard errors, log-transformed models, and logit-transformed models were compared (Supplementary Table S10).

The incremental association of treadmill parameters with PREVENT-CVD risk remained statistically significant across all transformations (all *p* < 0.001), although the magnitude of ΔR^2^ varied: linear model ΔR^2^ = 0.060 (with HC3 robust SE), log-transformed ΔR^2^ = 0.026, and logit-transformed ΔR^2^ = 0.027.

Untransformed linear models showed significant residual non-normality (Shapiro–Wilk *p* < 0.001) and heteroscedasticity (Breusch–Pagan *p* < 0.001), supporting the use of log-transformed models as the primary analysis (Supplementary Figures S1 and S2).

#### 3.8.3. Extended Clinical Adjustment

When the base model was expanded to include resting systolic blood pressure, total and HDL cholesterol, eGFR, smoking status, hypertension, and diabetes mellitus, the incremental explained variance attributable to treadmill parameters was negligible and non-significant (ΔR^2^ = 0.0004, *p* = 0.386; Table 3). This finding indicates substantial overlap between the physiological information captured by exercise testing and the clinical variables already embedded in the PREVENT algorithm.

#### 3.8.4. Two-Stage Residual Analyses

Two-stage residual analyses, in which treadmill parameters were regressed on the residuals from the extended clinical base model, yielded consistent findings (Supplementary Table S7). Sensitivity analyses excluding extreme HRR30 values produced similar results and did not materially change model estimates (Supplementary Table S8).

#### 3.8.5. Distributional Shifts

Redistribution analysis based on quartiles of predicted PREVENT-CVD risk showed that 14.2% of participants (55/387) shifted to a different estimated risk quartile after incorporation of treadmill parameters in the log-transformed model (Table 5). These distributional shifts should be interpreted as changes in the ranking of calculated risk estimates rather than formal clinical reclassification, as the dependent variable represents PREVENT-estimated risk rather than adjudicated cardiovascular events.

#### 3.8.6. Sex-Specific Analyses

Sex-stratified models (Table 6) showed significant incremental associations in both men (ΔR^2^ = 0.022, *p* < 0.001) and women (ΔR^2^ = 0.038, *p* < 0.001) when using the demographic base model.

#### 3.8.7. Model Statistics

The performance and incremental explained variance of the sex-stratified models are summarized in Table 7.

#### 3.8.8. Effect Sizes

Effect size estimates are summarized in Supplementary Table S9. Cohen’s f^2^ for the overall demographic base model was 0.154, corresponding to a medium effect size.

## 4. Discussion

This study examined the association between treadmill exercise testing (TET)-derived physiological parameters and 10-year cardiovascular risk as estimated by the 2023 American Heart Association PREVENT equations [4,5]. Because the dependent variable in our analyses is PREVENT-estimated risk rather than adjudicated cardiovascular events, the findings quantify the degree to which exercise-derived measures are associated with calculated risk estimates rather than their incremental prediction of clinical outcomes.

Our principal findings are twofold. First, when evaluated beyond basic demographic factors (age, sex, and BMI), several treadmill parameters—particularly double product, maximum METs, and heart rate recovery—showed a statistically significant association with PREVENT-estimated risk (ΔR^2^ = 0.026, *p* < 0.001 in log-transformed models). Second, when the full set of PREVENT input covariates was included in the base model, this incremental association became negligible (ΔR^2^ = 0.0004, *p* = 0.386), indicating that the physiological information captured by exercise testing substantially overlaps with the clinical variables already embedded in the PREVENT algorithm.

These findings suggest that treadmill parameters reflect the same underlying biological processes that drive PREVENT-estimated risk cardiovascular aging, hemodynamic burden, and metabolic dysregulation, but capture these processes through dynamic, stress-evoked responses rather than resting measurements. This perspective highlights the broader importance of dynamic cardiovascular assessment, given the complex interplay between cardiac function, hemodynamic load, and cardiopulmonary responses in cardiovascular disease [14].

The disappearance of independent associations after adjustment for the full PREVENT covariate set requires careful interpretation. PREVENT scores are deterministically computed from clinical inputs including resting blood pressure, lipids, renal function, glycemic status, and smoking. When these variables are entered as covariates, the base model reproduces approximately 97% of the variance in the PREVENT output, leaving minimal residual variance for additional predictors to explain. This near-complete recapitulation represents a statistical ceiling effect inherent to modeling a deterministic equation with its own inputs [15], and does not imply that exercise testing lacks clinical value.

Rather, the pattern of results suggests that treadmill-derived parameters and PREVENT input variables capture overlapping constructs through different physiological windows. Double product, for example, reflects the integrated hemodynamic response to graded physical stress, which is strongly influenced by the same vascular and autonomic factors that determine resting blood pressure—a core PREVENT input. Similarly, exercise capacity (METs) correlates with metabolic health parameters such as BMI and HbA1c that are themselves included in PREVENT. The clinical value of exercise testing may therefore lie not in providing statistically independent information beyond PREVENT, but in revealing the dynamic physiological expression of risk factors that are measured only at rest in conventional algorithms.

While PREVENT captures risk factor burden at a single time point, exercise testing provides a provoked and integrative assessment of cardiovascular reserve. Two individuals with identical PREVENT scores may exhibit markedly different hemodynamic responses to stress, reflecting differences in vascular compliance, autonomic regulation, and cardiorespiratory fitness that are not fully captured by resting measurements. In this sense, exercise testing functions as a physiological “stress test” for the risk profile itself, translating abstract numerical risk estimates into observable cardiovascular responses [16,17]. Estimating longer-term or lifetime risk, rather than relying exclusively on 10-year algorithms [18,19], may further clarify the role of exercise-derived markers in cardiovascular risk assessment.

The choice of outcome transformation substantially influenced both the magnitude of ΔR^2^ and the significance patterns of individual predictors. PREVENT risk estimates in our cohort were markedly right-skewed (skewness = 2.42), and untransformed linear regression produced non-normal residuals (Shapiro–Wilk *p* < 0.001), significant heteroscedasticity (Breusch–Pagan *p* < 0.001), and negative predicted values for 12.4% of observations—an impossibility for a risk percentage.

Log-transformation corrected these violations and yielded a more conservative but methodologically appropriate estimate of the incremental association (ΔR^2^ = 0.026 versus 0.060 in untransformed models). The transformation also altered the apparent significance of individual predictors: the ST/HR index, which appeared highly significant in untransformed models (*p* < 0.001), lost significance after log transformation (*p* = 0.501), whereas HRR at 1 min became significant (*p* < 0.001). These shifts likely reflect biased standard errors in the untransformed models due to violated distributional assumptions. The sensitivity of predictor-level inference to outcome transformation highlights the importance of verifying regression assumptions when modeling bounded and skewed risk scores as dependent variables.

Among all exercise variables, double product (maximum SBP × maximum HR) demonstrated the strongest and most consistent association with PREVENT-estimated risk. Although traditionally interpreted as a surrogate for myocardial oxygen consumption [20], in a screening population without inducible ischemia its association with cardiovascular risk more likely reflects the hemodynamic pressor response to exercise—a phenotype linked to arterial stiffness, impaired vascular compliance, and early target-organ damage [21]. Longitudinal studies have shown that exaggerated exercise blood pressure responses are associated with incident hypertension and adverse cardiovascular outcomes [22], suggesting that stress-evoked hemodynamic responses may reveal vascular abnormalities not fully apparent in resting measurements.

Double product is also influenced by factors beyond intrinsic vascular health, including age, sex, body composition, anxiety, and exercise protocol characteristics [23]. In our dataset, its association with PREVENT risk partly reflects the shared dependence of both measures on blood pressure physiology: PREVENT incorporates resting SBP and antihypertensive therapy, whereas double product captures the dynamic SBP response during exertion. This overlap was confirmed in the extended clinical adjustment model, where double product was no longer independently associated with risk estimates after adjustment for resting SBP and other PREVENT inputs.

Exercise capacity (METs) was inversely associated with PREVENT-estimated risk in both unadjusted and demographically adjusted models. This is consistent with extensive epidemiological evidence linking cardiorespiratory fitness to long-term survival. Myers et al. demonstrated that exercise capacity is a stronger predictor of mortality than established risk factors [8], and subsequent meta-analyses have confirmed a dose-dependent inverse relationship, with each additional MET associated with approximately a 10–12% reduction in mortality risk [24,25,26]. This relationship persists even when fitness levels change over time [27].

Exercise capacity represents a highly integrative physiological measure, reflecting the coordinated function of the cardiovascular, pulmonary, and musculoskeletal systems alongside autonomic regulation [28,29]. Low exercise capacity may therefore signal early cardiometabolic vulnerability before abnormalities become detectable in routine laboratory testing.

Heart rate recovery is a well-established marker of parasympathetic reactivation and has been consistently associated with cardiovascular mortality in population-based studies [9,30]. In our untransformed models, HRR at 1 min was not statistically significant. However, after correcting distributional violations through log transformation of the outcome variable, HRR emerged as independently associated with PREVENT-estimated risk.

This observation again highlights the importance of appropriate statistical modeling when working with skewed risk distributions. Impaired autonomic recovery likely reflects systemic autonomic dysregulation associated with metabolic syndrome and cardiovascular disease [23].

Although the ST/HR index did not reach statistical significance in log-transformed models, it has been shown to offer greater sensitivity for detecting ischemic burden than absolute ST-segment changes alone [31]. Subclinical coronary microvascular dysfunction may exist on a physiological continuum even in individuals with negative conventional exercise tests [32]. In the present cohort, however, ST-segment deviations were small by design, limiting the informative range of this parameter. The loss of significance after log transformation—despite apparent significance in untransformed models—further suggests that the initial finding was driven by distributional artifacts rather than a true physiological association.

The magnitude of associations varied across risk strata. In individuals with very low estimated risk (<1%), treadmill parameters did not contribute additional explained variance, whereas in intermediate and higher risk groups the associations were substantially stronger. This gradient suggests that exercise-derived physiological information may be most informative in populations where risk factor burden is present but risk quantification remains uncertain—precisely the clinical setting in which additional data are most needed for decision-making.

Sex-stratified analyses showed significant incremental associations in both men and women, with a numerically greater contribution observed in women. This pattern is consistent with prior observations of sex-related differences in hemodynamic and autonomic responses to exercise [33,34,35].

Redistribution analysis indicated that 14.2% of participants shifted to a different estimated risk quartile after incorporation of treadmill parameters. Because the dependent variable represents PREVENT-estimated risk rather than adjudicated outcomes, these shifts should be interpreted as changes in the ranking of calculated risk estimates rather than formal clinical reclassification.

Nevertheless, exercise testing may improve the communication and interpretation of cardiovascular risk. Individuals with identical PREVENT scores may display markedly different physiological responses during exercise, and these observable differences may enhance patient engagement and support shared decision-making regarding preventive therapies [16,17].

### Limitations

Several limitations should be considered. First, the dependent variable in all analyses was PREVENT-estimated risk rather than adjudicated cardiovascular events. Accordingly, the present analyses quantify associations with calculated risk estimates rather than improvements in outcome prediction.

Second, this single-center study included a relatively young and predominantly healthy screening population with low overall estimated cardiovascular risk, which may limit generalizability to older or higher-risk populations.

Third, HbA1c was unavailable for 22% of participants, reducing the sample size in some extended models. Although sensitivity analyses produced consistent findings, the potential impact of missingness should be acknowledged.

Fourth, the subgroup risk thresholds used in the present analyses were study-defined and have not been formally validated as PREVENT-specific clinical decision thresholds.

Finally, the cross-sectional design precludes causal inference. Prospective longitudinal studies incorporating adjudicated cardiovascular outcomes are required to determine whether exercise-derived physiological measures improve risk prediction beyond established algorithms.

## 5. Conclusions

Treadmill exercise testing parameters, particularly double product, exercise capacity (METs), and heart rate recovery are significantly associated with PREVENT-estimated cardiovascular risk beyond basic demographic factors. However, these associations substantially overlap with the clinical variables already incorporated in the PREVENT equations. Rather than providing statistically independent incremental information, exercise testing appears to capture the dynamic physiological expression of the same cardiovascular risk factors measured at rest by PREVENT.

The strongest associations were observed among individuals with intermediate estimated risk and in those aged ≥45 years, suggesting that exercise-derived physiological data may be most informative in populations where risk quantification remains uncertain. These findings support the potential role of treadmill testing as a physiological complement to conventional risk estimation, although prospective studies with adjudicated cardiovascular outcomes are required before clinical implementation.

## Figures and Tables

**Table 1:** Baseline Characteristics of the Study Population.

Parameter	Value
**Demographic Characteristics**	
Age, years	42.9 ± 9.0
Male sex, *n* (%)	222 (56.8)
Body mass index, kg/m^2^	27.3 ± 3.5
Current smoking, *n* (%)	143 (36.6)
Hypertension, *n* (%)	31 (7.9)
Hyperlipidemia, *n* (%)	8 (2.0)
Diabetes mellitus, *n* (%)	12 (3.1)
**Biochemical Parameters**	
Total cholesterol, mg/dL	186 ± 35
LDL cholesterol, mg/dL	104 ± 32
HDL cholesterol, mg/dL	48.0 [41.0–56.0]
HbA1c, %	5.60 [5.40–5.90]
eGFR, mL/min/1.73 m^2^	103.8 ± 12.6
**Exercise Parameters**	
Maximum METs	12.0 [9.4–14.8]
Maximum heart rate, bpm	161 ± 15.1
Resting heart rate, bpm	88.0 [78.0–96.0]
Maximum systolic blood pressure, mmHg	150.0 [139.0–164.0]
Resting systolic blood pressure, mmHg	120.0 [114.5–131.0]
HRR, 30 s	8.0 [4.0–13.0]
HRR, 1 min	28.0 [21.0–36.0]
HRR, 2 min	50.0 [42.0–59.0]
Maximum ST/HR index	0.70 [0.30–1.10]
Double product (HR × SBP, ×10^3^)	20.0 ± 3.0
**PREVENT Risk Scores, %**	
CVD	1.8 [0.9–3.5]
ASCVD	1.2 [0.6–2.3]
HF	0.6 [0.3–1.4]

Note: Data are presented as mean ± standard deviation, median [interquartile range], or *n* (%). Bold text indicates category subheadings. Abbreviations: ASCVD, atherosclerotic cardiovascular disease; CVD, cardiovascular disease; eGFR, estimated glomerular filtration rate; HbA1c, glycated hemoglobin; HDL, high-density lipoprotein; HRR, heart rate recovery; LDL, low-density lipoprotein; METs, metabolic equivalents; SBP, systolic blood pressure; ST/HR, ST-segment/heart rate.

**Table 2:** Hierarchical Regression Analysis for Log-Transformed PREVENT-CVD Risk (Demographic Base Model).

Variable	Model 0 β	Model 0 SE	Model 0 *p*	Model 1 β	Model 1 SE	Model 1 *p*	Std β (Model 1)
Age, years	0.0889	0.0025	<0.001	0.0851	0.0025	<0.001	0.777
Male sex	0.6025	0.0453	<0.001	0.6153	0.0493	<0.001	0.310
BMI, kg/m^2^	0.0314	0.0065	<0.001	0.0169	0.0065	0.009	0.060
Maximum METs	—	—	—	−0.0357	0.0116	0.002	−0.079
Double product (×10^3^)	—	—	—	0.0385	0.0074	<0.001	0.116
Maximum ST/HR index	—	—	—	0.0191	0.0283	0.500	0.015
HRR (1 min)	—	—	—	−0.00676	0.00182	<0.001	−0.081

Note. Model 0 included age, sex, and body mass index. Model 1 additionally included treadmill derived parameters (maximum METs, double product, ST/HR index, and HRR at 1 min). Model 0: R^2^ = 0.805, adjusted R^2^ = 0.804. Model 1: R^2^ = 0.831, adjusted R^2^ = 0.828, ΔR^2^ = 0.026, F-change = 14.58, *p* < 0.001. Abbreviations: BMI, body mass index; HRR, heart rate recovery; METs, metabolic equivalents; ST/HR, ST-segment/heart rate.

**Table 3:** Hierarchical Regression Analysis for Log-Transformed PREVENT-CVD Risk (Extended Clinical Base Model).

Variable	Model 0 β	Model 0 SE	Model 0 *p*	Model 1 β	Model 1 SE	Model 1 *p*	Std β (Model 1)
Age, years	0.0767	0.00141	<0.001	0.0771	0.00147	<0.001	0.704
Male sex	0.2920	0.0224	<0.001	0.2786	0.0253	<0.001	0.140
BMI, kg/m^2^	0.00819	0.00295	0.0058	0.00917	0.00302	0.0025	0.033
Resting SBP, mmHg	0.01497	0.00073	<0.001	0.01530	0.00105	<0.001	0.228
Total cholesterol, mg/dL	0.00265	0.00029	<0.001	0.00267	0.00029	<0.001	0.095
HDL cholesterol, mg/dL	−0.01522	0.00095	<0.001	−0.01528	0.00096	<0.001	−0.187
eGFR, mL/min/1.73 m^2^	−0.00353	0.00094	<0.001	−0.00338	0.00095	<0.001	−0.043
Current smoking	0.5448	0.0208	<0.001	0.5482	0.0210	<0.001	0.268
Hypertension	0.3228	0.0397	<0.001	0.3272	0.0402	<0.001	0.090
Diabetes mellitus	0.6662	0.0594	<0.001	0.6690	0.0611	<0.001	0.118
Maximum METs	—	—	—	0.00518	0.00549	0.346	0.011
Double product (×10^3^)	—	—	—	−0.0021	0.0047	0.652	−0.006
Maximum ST/HR index	—	—	—	−0.02259	0.01301	0.083	−0.017
HRR (1 min)	—	—	—	−0.00042	0.00085	0.623	−0.005

Note. Model 0 included the full set of available PREVENT input covariates (age, sex, body mass index, resting systolic blood pressure, total cholesterol, HDL cholesterol, eGFR, current smoking, hypertension, and diabetes mellitus). Model 1 additionally included treadmill-derived parameters (maximum METs, double product, ST/HR index, and HRR at 1 min). Model 0: R^2^ = 0.966, adjusted R^2^ = 0.965. Model 1: R^2^ = 0.966, adjusted R^2^ = 0.965, ΔR^2^ = 0.0004, F-change = 1.04, *p* = 0.386. Abbreviations: BMI, body mass index; eGFR, estimated glomerular filtration rate; HDL, high-density lipoprotein; HRR, heart rate recovery; METs, metabolic equivalents; SBP, systolic blood pressure; ST/HR, ST-segment/heart rate.

**Table 4:** Incremental Explained Variance (ΔR^2^) of Treadmill-Derived Parameters Across Clinically Relevant Subgroups.

Stratification	Group	*n*	ΔR^2^	*p* Value
Estimated risk (PREVENT-CVD)	<1%	100	0.0086	0.794
	1–5%	221	0.0522	<0.001
	≥5%	66	0.2457	<0.001
Risk factor burden	0 RF	217	0.0228	<0.001
	≥1 RF	170	0.0503	<0.001
Age group	<45 years	229	0.0306	<0.001
	≥45 years	158	0.1131	<0.001
Sex	Male	219	0.0222	<0.001
	Female	168	0.0378	<0.001

Note. ΔR^2^ represents the increase in explained variance after adding treadmill-derived parameters (maximum METs, double product, ST/HR index, and HRR at 1 min) to a base model adjusted for age, sex, and body mass index (sex excluded from the base model in sex-stratified analyses).

**Table 5:** Estimated Risk Quartile Redistribution Matrix.

Base Quartile	Q1	Q2	Q3	Q4
Q1	91	7	0	0
Q2	6	78	12	0
Q3	0	12	75	9
Q4	0	0	9	88

Note. Values represent the number of participants whose estimated PREVENT-CVD risk quartile changed after inclusion of treadmill-derived parameters in the regression model. Rows indicate baseline quartiles, and columns indicate quartiles after incorporation of exercise variables.

**Table 6:** Sex-Specific Hierarchical Regression Models for Log-Transformed PREVENT-CVD Risk.

Sex	Predictor	*n*	β	SE	Std β	*p* Value
Male	Model summary	219	—	—	—	—
	Maximum METs		−0.0219	0.0144	−0.0460	0.129
	Double product		0.00394	0.0009	0.1305	<0.001
	ST/HR index		0.0511	0.0322	0.0473	0.114
	HRR (1 min)		−0.00470	0.0024	−0.0567	0.056
Female	Model summary	168	—	—	—	—
	Maximum METs		−0.0425	0.0182	−0.0840	0.021
	Double product		0.00391	0.0011	0.1179	<0.001
	ST/HR index		−0.00325	0.0502	−0.00217	0.948
	HRR (1 min)		−0.00815	0.0026	−0.1059	0.003

Note: Abbreviations: SE, standard error; Std β, standardized beta coefficient.

**Table 7:** Model Statistics for Sex-Specific Hierarchical Regression Analysis.

Sex	*n*	Base R^2^	Adjusted R^2^	Model R^2^	Adjusted Model R^2^	ΔR^2^	F-Change	*p* Value
Male	219	0.803	0.801	0.825	0.820	0.022	6.71	<0.001
Female	168	0.785	0.782	0.822	0.816	0.038	8.57	<0.001

Note. Sex-stratified hierarchical regression models were constructed using log-transformed PREVENT-CVD risk as the dependent variable. Base models included age and body mass index (sex excluded). Exercise-derived variables (maximum METs, double product, ST/HR index, and HRR at 1 min) were subsequently added to assess incremental explained variance.

## Data Availability

The data supporting the findings of this study are available from the corresponding author upon reasonable request.

## Supplementary Materials

The following supporting information can be downloaded at: https://www.mdpi.com/article/10.3390/jcm15062346/s1. Table S1: Correlations Between Treadmill Parameters and PREVENT-CVD Risk. Table S2: Sequential Hierarchical Models Examining the Association of Individual Exercise Parameters With Log-Transformed PREVENT Risk Estimates. Table S3: Quartile-Based Sensitivity Analysis for Log-Transformed PREVENT-CVD Risk. Table S4A: Exercise Parameters Across Clinical Subgroups. Table S4B: PREVENT Risk Scores Across Clinical Subgroups. Table S5: Correlations Between Treadmill Parameters and Traditional Cardiovascular Risk Factors. Table S6A: Bootstrap Validation Summary. Table S6B: Bootstrap 95% Confidence Intervals for Treadmill Coefficients. Table S7: Two-Stage Residual Analysis for Log-Transformed PREVENT-CVD Risk. Table S8: Sensitivity Analysis of HRR30 After Exclusion of Extreme Values. Table S9: Effect size estimates (Cohen’s f^2^). Supplementary Table S10. Sensitivity Analysis Across Outcome Transformations for PREVENT-CVD Risk (Demographic Base Model). Figure S1. Regression diagnostic plots for the full treadmill-augmented model (Model 1) with untransformed PREVENT-CVD risk (%). Figure S2. Regression diagnostic plots for the full treadmill-augmented model (Model 1) with log-transformed PREVENT-CVD risk.

## Abbreviations

The following abbreviations are used in this manuscript:

AHA	American Heart Association
ASCVD	Atherosclerotic Cardiovascular Disease
BMI	Body Mass Index
CVD	Cardiovascular Disease
eGFR	Estimated Glomerular Filtration Rate
HbA1c	Glycated Hemoglobin
HF	Heart Failure
HR	Heart Rate
HRR	Heart Rate Recovery
LDL	Low-Density Lipoprotein
METs	Metabolic Equivalents
PREVENT	Predicting Risk of Cardiovascular Disease Events
SBP	Systolic Blood Pressure
ST/HR	ST-Segment/Heart Rate Index
TET	Treadmill Exercise Testing
VIF	Variance Inflation Factor

## References

[1] Mensah G.A., Fuster V., Murray C.J.L., Roth G.A. (2023). Global burden of cardiovascular diseases and risks, 1990–2022. J. Am. Coll. Cardiol..

[2] Erbel R., Delaney J.A.C., Lehmann N., McClelland R.L., Möhlenkamp S., Kronmal R.A., Schmermund A., Moebus S., Dragano N., Stang A. (2008). Signs of subclinical coronary atherosclerosis in relation to risk factor distribution in the Multi-Ethnic Study of Atherosclerosis (MESA) and the Heinz Nixdorf Recall Study (HNR). Eur. Heart J..

[3] Ibanez B., Fernández-Ortiz A., Fernández-Friera L., García-Lunar I., Andrés V., Fuster V. (2021). Progression of Early Subclinical Atherosclerosis Study: JACC Focus Seminar 7/8. J. Am. Coll. Cardiol..

[4] Khan S.S., Matsushita K., Sang Y., Ballew S.H., Grams M.E., Surapaneni A., Moebus S., Dragano N., Stang A., Jöckel K.H. (2024). Development and Validation of the American Heart Association’s PREVENT Equations. Circulation.

[5] Brett A.S. (2024). A New Cardiovascular Risk Calculator from the American Heart Association. NEJM J. Watch.

[6] Murphy B.S., Hershey M.S., Huang S., Nam Y., Post W.S., McClelland R.L., DeFilippis A.P. (2025). PREVENT Risk Score vs the Pooled Cohort Equations in MESA. JACC Adv..

[7] Bouque J.M., Charlton G.T., Holland B.H., Belyea C.M., Watson D.D., Beller G.A. (2011). Prognosis in patients achieving ≥10 METS on exercise stress testing: Was SPECT imaging useful?. J. Nucl. Cardiol..

[8] Myers J., Prakash M., Froelicher V., Do D., Partington S., Atwood J.E. (2002). Exercise capacity and mortality among men referred for exercise testing. N. Engl. J. Med..

[9] Cole C.R., Blackstone E.H., Pashkow F.J., Snader C.E., Lauer M.S. (1999). Heart-rate recovery immediately after exercise as a predictor of mortality. N. Engl. J. Med..

[10] Qiu S., Cai X., Sun Z., Li L., Zuegel M., Steinacker J.M., Schumann U. (2017). Heart Rate Recovery and Risk of Cardiovascular Events and All-Cause Mortality: A Meta-Analysis of Prospective Studies. J. Am. Heart Assoc..

[11] Alashi A., Riaz H., Lang R., Seballos R., Feinleib S., Sukol R., Cho L., Cremer P., Jaber W., Griffin B.P. (2019). Incremental Prognostic Value of Exercise Stress Testing in Primary Prevention. Am. J. Cardiol..

[12] Balady G.J., Larson M.G., Vasan R.S., Leip E.P., O’Donnell C.J., Levy D. (2004). Usefulness of exercise testing in the prediction of coronary disease risk among asymptomatic persons as a function of the Framingham risk score. Circulation.

[13] Gielen S., Laughlin M.H., O’Conner C., Duncker D.J. (2015). Exercise training in patients with heart disease: Review of beneficial effects and clinical recommendations. Prog. Cardiovasc. Dis..

[14] Tetaj N., Capecchi G., Rubino D., Stazi G.V., Cingolani E., Lesci A., Segreti A., Grigioni F., Bocci M.G. (2026). Respiratory Support in Cardiogenic Pulmonary Edema: Clinical Insights from Cardiology and Intensive Care. J. Cardiovasc. Dev. Dis..

[15] Harrell F.E. (2001). Regression Modeling Strategies: With Applications to Linear Models, Logistic Regression, and Survival Analysis.

[16] Goff D.C., Lloyd-Jones D.M., Bennett G., Coady S., D’Agostino R.B., Gibbons R., Greenland P., Lackland D.T., Levy D., O’Donnell C.J. (2014). 2013 ACC/AHA guideline on the assessment of cardiovascular risk: A report of the American College of Cardiology/American Heart Association Task Force on Practice Guidelines. J. Am. Coll. Cardiol..

[17] Arnett D.K., Blumenthal R.S., Albert M.A., Buroker A.B., Goldberger Z.D., Hahn E.J., Himmelfarb C.D., Khera A., Lloyd-Jones D., McEvoy J.W. (2019). 2019 ACC/AHA Guideline on the Primary Prevention of Cardiovascular Disease: A Report of the American College of Cardiology/American Heart Association Task Force on Clinical Practice Guidelines. Circulation.

[18] Pencina M.J., D’Agostino R.B., Larson M.G., Massaro J.M., Vasan R.S. (2009). Predicting the 30-year risk of cardiovascular disease: The framingham heart study. Circulation.

[19] Lloyd-Jones D.M., Leip E.P., Larson M.G., D’Agostino R.B., Beiser A., Wilson P.W.F., Wolf P.A., Levy D. (2006). Prediction of lifetime risk for cardiovascular disease by risk factor burden at 50 years of age. Circulation.

[20] Jorgensen C.R., Wang K., Wang Y., Gobel F.L., Nelson R.R., Taylor H. (1973). Effect of propranolol on myocardial oxygen consumption and its hemodynamic correlates during upright exercise. Circulation.

[21] Schultz M.G., Picone D.S., Nikolic S.B., Williams A.D., Sharman J.E. (2016). Exaggerated blood pressure response to early stages of exercise stress testing and presence of hypertension. J. Sci. Med. Sport.

[22] Kunimatsu N., Tsukamoto H., Ogoh S. (2024). Exaggerated Blood Pressure Response to Exercise Is a Risk of Future Hypertension Even in Healthy, Normotensive Young Individuals—Potential Preventive Strategies for This Phenomenon?. J. Clin. Med..

[23] Buchheit M., Gindre C. (2006). Cardiac parasympathetic regulation: Respective associations with cardiorespiratory fitness and training load. Am. J. Physiol. Heart Circ. Physiol..

[24] Wen C.P., Wai J.P., Tsai M.K., Yang Y.C., Cheng T.Y., Lee M.C., Chan H.T., Tsao C.K., Tsai S.P., Wu X. (2011). Minimum amount of physical activity for reduced mortality and extended life expectancy: A prospective cohort study. Lancet.

[25] Al-Mallah M.H., Sakr S., Al-Qunaibet A. (2018). Cardiorespiratory Fitness and Cardiovascular Disease Prevention: An Update. Curr. Atheroscler. Rep..

[26] Kodama S., Saito K., Tanaka S., Maki M., Yachi Y., Asumi M., Sugawara A., Totsuka K., Shimano H., Ohashi Y. (2009). Cardiorespiratory fitness as a quantitative predictor of all-cause mortality and cardiovascular events in healthy men and women: A meta-analysis. JAMA.

[27] Ehrman J.K., Brawner C.A., Al-Mallah M.H., Qureshi W.T., Blaha M.J., Keteyian S.J. (2017). Cardiorespiratory Fitness Change and Mortality Risk Among Black and White Patients: Henry Ford Exercise Testing (FIT) Project. Am. J. Med..

[28] Epelde F. (2024). Impact of Exercise on Physiological, Biochemical, and Analytical Parameters in Patients with Heart Failure with Reduced Ejection Fraction. Medicina.

[29] Pliouta L., Lampsas S., Kountouri A., Korakas E., Thymis J., Kassi E., Oikonomou E., Ikonomidis I., Lambadiari V. (2025). Mitochondrial Dysfunction in the Development and Progression of Cardiometabolic Diseases: A Narrative Review. J. Clin. Med..

[30] Nishime E.O., Cole C.R., Blackstone E.H., Pashkow F.J., Lauer M.S. (2000). Heart rate recovery and treadmill exercise score as predictors of mortality in patients referred for exercise ECG. JAMA.

[31] Carlén A., Nylander E., Åström Aneq M., Gustafsson M. (2019). ST/HR variables in firefighter exercise ECG—Relation to ischemic heart disease. Physiol. Rep..

[32] Taqueti V.R., Di Carli M.F. (2018). Coronary Microvascular Disease Pathogenic Mechanisms and Therapeutic Options: JACC State-of-the-Art Review. J. Am. Coll. Cardiol..

[33] Rascón J., Trujillo E., Morales-Acuña F., Gurovich A.N. (2020). Differences between Males and Females in Determining Exercise Intensity. Int. J. Exerc. Sci..

[34] Arena R., Arrowood J.A., Fei D., Shelar S., Helm S., Kraft K.A. (2010). The influence of sex on the relationship between heart rate recovery and other cardiovascular risk factors in apparently healthy subjects. Scand. J. Med. Sci. Sports.

[35] Liu C.C., Kuo T.B.J., Yang C.C.H. (2003). Effects of estrogen on gender-related autonomic differences in humans. Am. J. Physiol. Heart Circ. Physiol..

