# Supplementary material for: Association of Treadmill Exercise Testing Parameters with PREVENT-Estimated Cardiovascular Risk: A Cross-Sectional Analysis
Source: J Clin Med. 2026 Mar 19;15(6):2346. doi: 10.3390/jcm15062346 (PMC13026620; doi:10.3390/jcm15062346)
Supplement: Supplementary file 1 [file jcm-15-02346-s001.zip › jcm-4175210-supplementary.pdf]

Supplementary Table S1. Correlations Between Treadmill Parameters and PREVENT-CVD Risk

Parameter	Unadjusted r	Unadjusted p	Adjusted r†	Adjusted p
HRR (1 min)	−0.314	<0.001	−0.092	0.072
HRR (2 min)	−0.342	<0.001	−0.129	0.011
Maximum METs	−0.042	0.408	−0.219	<0.001
ST/HR index	0.055	0.277	0.185	<0.001
Double product (×10^3)	0.260	<0.001	0.278	<0.001

Note. Adjusted correlations were calculated using partial correlation after adjustment for age, sex, and body mass index.

Supplementary Table S2. Sequential Hierarchical Models Examining the Association of Individual Exercise Parameters With Log-Transformed PREVENT Risk Estimates

Outcome	Step	R^2	ΔR^2	F-change	p value
PREVENT-CVD	Base	0.805	—	—	—
	Maximum METs	0.810	0.0049	9.81	0.0019
	ST/HR index	0.810	0.0001	0.22	0.643
	Double product	0.825	0.0149	32.32	<0.001
PREVENT-ASCVD	Base	0.786	—	—	—
	Maximum METs	0.793	0.0061	11.24	<0.001
	ST/HR index	0.793	0.00007	0.13	0.718
	Double product	0.814	0.0210	42.98	<0.001
PREVENT-HF	Base	0.812	—	—	—
	Maximum METs	0.816	0.0039	8.15	0.0045
	Double product	0.825	0.0094	20.53	<0.001
	ST/HR index	0.825	0.00011	0.24	0.624

Note. Base models included age, sex, and body mass index. Exercise parameters were added sequentially in the order shown to examine their association with log-transformed PREVENT risk estimates.

Supplementary Table S3. Quartile-Based Sensitivity Analysis for Log-Transformed PREVENT-CVD Risk

Quartile	Min (%)	Max (%)	n	ΔR^2	p value
Q1	0.1	0.9	100	0.0086	0.794
Q2	1.0	1.8	96	0.0345	0.430
Q3	1.9	3.5	94	0.1079	0.0088
Q4	3.6	19.5	97	0.1678	<0.001

Note. Quartile-based sensitivity analysis examining the incremental explained variance (ΔR^2) after adding treadmill-derived parameters (maximum METs, double product, ST/HR index, and HRR at 1 minute) to the demographic base model (age, sex, and body mass index).

Supplementary Table S4A. Exercise Parameters Across Clinical Subgroups

Parameter	Subgroup	Values	p value
HRR (30 s)	Male / Female	8.0 [4.0–12.0] / 9.0 [5.0–14.0]	0.124
	Non-smoker / Smoker	8.0 [4.0–13.2] / 8.0 [5.0–12.0]	0.877
	No HT / HT	8.0 [4.0–13.0] / 7.0 [3.5–11.0]	0.107
	No DM / DM	8.0 [4.0–13.0] / 6.5 [0.0–10.0]	0.096
	No Dys / Dys	8.0 [4.0–13.0] / 7.0 [3.5–10.5]	0.360
HRR (1 min)	Male / Female	27.0 [20.0–33.0] / 30.0 [22.0–39.0]	0.0016
	Non-smoker / Smoker	29.0 [21.0–37.2] / 27.0 [19.5–33.0]	0.0248
	No HT / HT	29.0 [21.0–36.2] / 24.0 [18.0–28.0]	0.0031
	No DM / DM	28.0 [21.0–36.0] / 23.0 [17.5–27.2]	0.030
	No Dys / Dys	28.0 [21.0–36.0] / 24.5 [17.5–30.0]	0.254
HRR (2 min)	Male / Female	50.0 [42.2–57.8] / 52.0 [40.0–60.0]	0.136
	Non-smoker / Smoker	51.0 [42.0–59.0] / 50.0 [42.0–56.0]	0.190
	No HT / HT	51.0 [43.0–59.0] / 40.0 [36.0–50.5]	<0.001
	No DM / DM	51.0 [42.0–59.0] / 39.0 [32.0–49.0]	0.0019
	No Dys / Dys	50.0 [42.0–59.0] / 39.0 [35.0–50.5]	0.0228

Parameter	Subgroup	Values	p value
Maximum HR	Male / Female	162.4 ± 14.8 / 159.3 ± 15.4	0.046
	Non-smoker / Smoker	161.2 ± 15.1 / 160.8 ± 15.1	0.786
	No HT / HT	161.8 ± 15.0 / 152.6 ± 14.0	0.0014
	No DM / DM	161.2 ± 15.0 / 156.9 ± 17.4	0.418
	No Dys / Dys	161.1 ± 15.0 / 158.8 ± 18.1	0.725
Maximum METs	Male / Female	12.0 [9.4–14.8] / 9.4 [9.4–12.0]	<0.001
	Non-smoker / Smoker	12.0 [9.4–12.0] / 12.0 [10.7–12.0]	0.007
	No HT / HT	12.0 [9.4–12.0] / 9.4 [8.2–12.0]	0.005
	No DM / DM	12.0 [9.4–12.0] / 8.2 [6.9–12.0]	0.0012
	No Dys / Dys	12.0 [9.4–12.0] / 6.9 [6.4–10.1]	0.0019
ST/HR index	Male / Female	0.60 [0.20–1.00] / 0.80 [0.40–1.20]	0.006
	Non-smoker / Smoker	0.70 [0.30–1.10] / 0.70 [0.30–1.20]	0.314
	No HT / HT	0.70 [0.30–1.10] / 0.70 [0.25–1.70]	0.505
	No DM / DM	0.70 [0.30–1.10] / 0.70 [0.15–1.10]	0.804
	No Dys / Dys	0.70 [0.30–1.10] / 0.55 [0.00–1.77]	0.805
Double product ($\times10^3$)	Male / Female	203.6 ± 28.6 / 196.2 ± 30.7	0.0156
	Non-smoker / Smoker	202.8 ± 29.5 / 196.2 ± 29.7	0.0358
	No HT / HT	199.6 ± 29.3 / 209.1 ± 33.9	0.140
	No DM / DM	199.9 ± 29.0 / 213.9 ± 47.4	0.333
	No Dys / Dys	200.0 ± 29.2 / 217.1 ± 47.9	0.349

Note. Continuous variables are presented as **median [interquartile range] or mean ± standard deviation**, depending on distribution.

Supplementary Table S4B. PREVENT Risk Scores Across Clinical Subgroups

Risk Score	Subgroup	Values	p value
PREVENT-CVD (%)	Male / Female	2.20 [1.30–4.45] / 1.25 [0.57–2.40]	<0.001
	Non-smoker / Smoker	1.50 [0.70–2.90] / 2.15 [1.40–4.50]	<0.001

Risk Score	Subgroup	Values	p value
	No HT / HT	1.70 [0.90–3.00] / 6.50 [3.70–10.05]	<0.001
	No DM / DM	1.70 [0.90–3.30] / 8.05 [6.20–11.62]	<0.001
	No Dys / Dys	1.70 [0.90–3.40] / 9.15 [6.15–11.78]	<0.001
PREVENT-ASCVD (%)	Male / Female	1.60 [0.90–2.90] / 0.80 [0.40–1.50]	<0.001
	Non-smoker / Smoker	1.00 [0.50–1.90] / 1.45 [1.00–2.88]	<0.001
	No HT / HT	1.10 [0.60–2.00] / 3.80 [2.45–5.95]	<0.001
	No DM / DM	1.20 [0.60–2.12] / 4.85 [3.25–6.80]	<0.001
	No Dys / Dys	1.20 [0.60–2.20] / 5.70 [3.42–6.98]	<0.001
PREVENT-HF (%)	Male / Female	0.70 [0.40–1.80] / 0.50 [0.20–1.00]	<0.001
	Non-smoker / Smoker	0.50 [0.20–1.20] / 0.70 [0.50–1.75]	<0.001
	No HT / HT	0.60 [0.30–1.18] / 3.10 [1.45–5.45]	<0.001
	No DM / DM	0.60 [0.30–1.30] / 5.45 [3.22–10.15]	<0.001
	No Dys / Dys	0.60 [0.30–1.30] / 5.75 [4.65–10.28]	<0.001

Note. Values are presented as median [interquartile range].

Supplementary Table S5. Correlations Between Treadmill Parameters and Traditional Cardiovascular Risk Factors

Exercise Parameter	Age (r)	Age p	Sex (r)	Sex p	HTN (r)	HTN p	DM (r)	DM p	BMI (r)	BMI p
Maximum METs	-0.144	0.004	0.485	<0.001	-0.142	0.0049	-0.164	0.0011	-0.286	<0.001
Double product	0.094	0.062	0.141	0.005	0.099	0.050	0.056	0.270	0.234	<0.001
ST/HR index	0.101	0.046	-0.138	0.006	0.034	0.505	-0.013	0.803	0.098	0.052
HRR (1 min)	-0.205	<0.001	-0.159	0.0016	-0.150	0.0030	-0.110	0.030	-0.055	0.277

Exercise Parameter	Age (r)	Age p	Sex (r)	Sex p	HTN (r)	HTN p	DM (r)	DM p	BMI (r)	BMI p
HRR (2 min)	-0.274	<0.001	-0.075	0.136	-0.204	<0.001	-0.157	0.0018	-0.047	0.354

Note. Values represent correlation coefficients (r) with corresponding p-values. Sex, hypertension, and diabetes were coded as binary variables for correlation analyses. HT:hypertension DM:diabetes mellitus BMI:body mass index

Supplementary Table S6A. Bootstrap Validation Summary (B = 1000)

Metric	Point Estimate	Bootstrap Mean	95% CI Lower	95% CI Upper
R^2 (Base)	0.805	0.807	0.771	0.841
R^2 (Full)	0.831	0.834	0.804	0.861
ΔR^2	0.026	0.027	0.014	0.045

Note. Bootstrap validation performed with 1000 resamples to assess stability of the hierarchical regression model estimates. Values are rounded for presentation.

Supplementary Table S6B. Bootstrap 95% Confidence Intervals for Treadmill Coefficients

Predictor	β	95% CI Lower	95% CI Upper
Maximum METs	-0.0357	-0.0586	-0.0127
Double product (×10^3)	0.00385	0.00239	0.00518
ST/HR index	0.0191	-0.0285	0.0721
HRR (1 min)	-0.00676	-0.01035	-0.00344

Note. Coefficients and confidence intervals were derived from bootstrap resampling (B = 1000) to evaluate the stability of treadmill-derived predictors in the hierarchical regression model. Values are rounded for presentation.

Supplementary Table S7. Two-Stage Residual Analysis for Log-Transformed PREVENT-CVD Risk

Stage	Outcome	Predictor Set	n	R^2	p value
Stage 1	ln(PREVENT-CVD)	Age, sex, BMI, resting SBP, total cholesterol, HDL cholesterol, eGFR, smoking, hypertension, diabetes	387	0.966	<0.001
Stage 2	Stage 1 residuals	Maximum METs, double product, ST/HR index, HRR (1 min)	387	0.009	0.480

Note. Stage 1 models the log-transformed PREVENT-CVD risk using the full set of PREVENT input variables. Stage 2 examines whether treadmill-derived parameters explain residual variance beyond the clinical predictors. Values are rounded for presentation.

Supplementary Table S8. Sensitivity Analysis of HRR30 After Exclusion of Extreme Values

Model	n	HRR30 β (Unstd.)	p value	Model R^2
Original full model	387	-0.00127	0.642	0.825
Outliers excluded	385	-0.00203	0.511	0.825

Note. Outlier limits were defined using the **3×IQR rule** for HRR30 (range: −23.0 to 40.0). Values are rounded for presentation.

Supplementary Table S9. Effect Sizes of Treadmill Parameters

Model	ΔR^2	R^2 (full)	Cohen's f^2
Overall	0.026	0.831	0.154
Male	0.022	0.825	0.127
Female	0.038	0.822	0.213
Extended clinical model	0.0004	0.966	0.011

Note. Effect sizes were calculated using Cohen's $f^2 = \Delta R^2 / (1 - R^2\_full)$ to quantify the magnitude of the incremental contribution of treadmill-derived parameters to the regression models. Values are rounded for presentation.

Supplementary Table S10. Sensitivity Analysis Across Outcome Transformations for PREVENT-CVD Risk (Demographic Base Model)

Variable	UTF β	UTF SE (HC3)	UTF Std β	UTF p (HC3)	Log-TF β	Log-TF SE	Log-TF Std β	Log-TF p	Logit-TF β	Logit-TF SE	Logit-TF Std β	Logit-TF p
Age, years	0.249	0.016	0.71	<0.001	0.085	0.002	0.777	<0.001	0.088	0.003	0.779	<0.001
Male sex	1.466	0.205	0.23	<0.001	0.615	0.049	0.310	<0.001	0.631	0.050	0.308	<0.001
BMI, kg/m^2	−0.060	0.028	−0.067	0.033	0.017	0.006	0.060	0.009	0.016	0.007	0.056	0.015
Maximum METs	−0.227	0.067	−0.156	<0.001	−0.036	0.012	−0.079	0.002	−0.038	0.012	−0.082	0.001
Double product (×10^3)	0.018	0.005	0.171	<0.001	0.004	0.001	0.116	<0.001	0.004	0.001	0.119	<0.001
ST/HR index	0.537	0.204	0.128	0.009	0.019	0.028	0.015	0.500	0.025	0.029	0.019	0.386
HRR (1 min)	−0.008	0.008	−0.031	0.317	−0.007	0.002	−0.081	<0.001	−0.007	0.002	−0.080	<0.001

Model summary

Metric	Untransformed	Log-transformed	Logit-transformed
Base R^2	0.639	0.805	0.807
Full R^2	0.699	0.831	0.834
ΔR^2	0.060	0.026	0.027

Metric	Untransformed	Log-transformed	Logit-transformed
F-change	18.84	14.58	15.21
p (F-change)	<0.001	<0.001	<0.001
Shapiro–Wilk p (residuals)	<0.001	0.305	0.220
Breusch–Pagan p	<0.001	<0.001	<0.001
Negative predictions, n (%)	48 (12.4%)	0	0

Note. UTF: Untransformed**,** Log-TF: Log-transformed Logit-TF: Logit-transformed

Base model included age, sex, and BMI. Treadmill parameters (maximum METs, double product, ST/HR index, HRR at 1 min) were added in Model 1. Untransformed models used heteroscedasticity-consistent (HC3) standard errors. Log-transformation: ln(PREVENT-CVD). Logit-transformation: logit(PREVENT-CVD/100). ΔR^2 indicates incremental explained variance attributable to treadmill parameters. Std β = standardized regression coefficient.

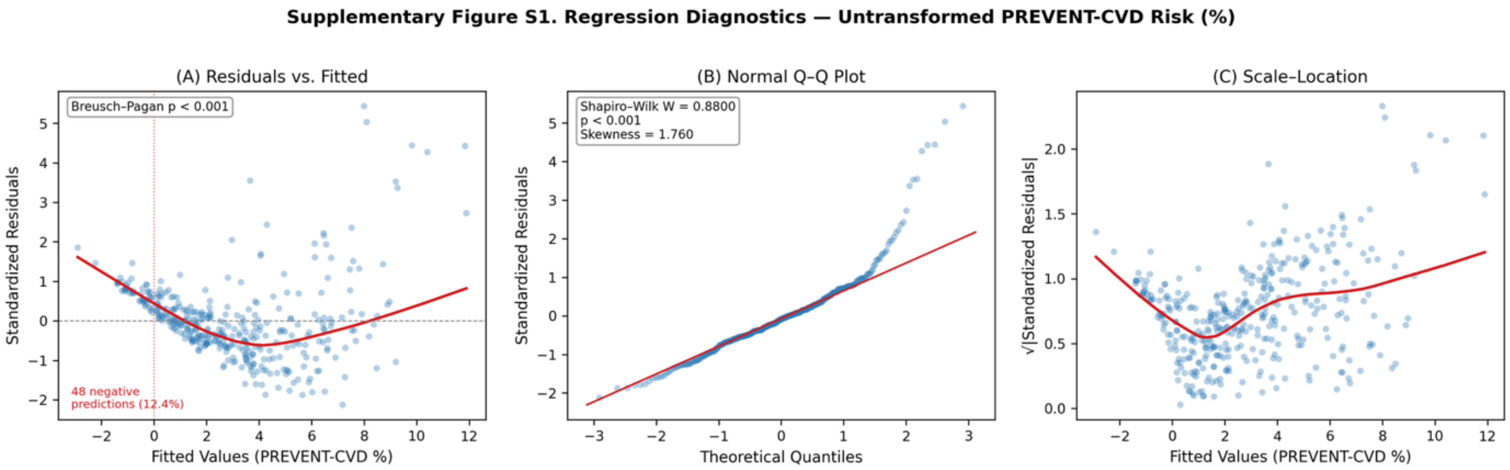

Supplementary Figure S1. Regression diagnostic plots for the full treadmill-augmented model (Model 1) with untransformed PREVENT-CVD risk (%) as the dependent variable (n = 387).

(A) Residuals vs. fitted values plot showing a prominent funnel-shaped pattern indicative of heteroscedasticity (Breusch–Pagan p < 0.001). The red dashed vertical line indicates the zero threshold;

48 observations (12.4%) had negative predicted values, which are impossible for a risk percentage. (B) Normal Q–Q plot demonstrating substantial right-tail deviation from normality (Shapiro–Wilk W = 0.880, p < 0.001; residual skewness = 1.76). (C) Scale–location plot confirming increasing residual variance with higher fitted values. These findings support the use of log-transformation as the primary analytical approach.

Supplementary Figure S2. Regression Diagnostics — Log-Transformed PREVENT-CVD Risk

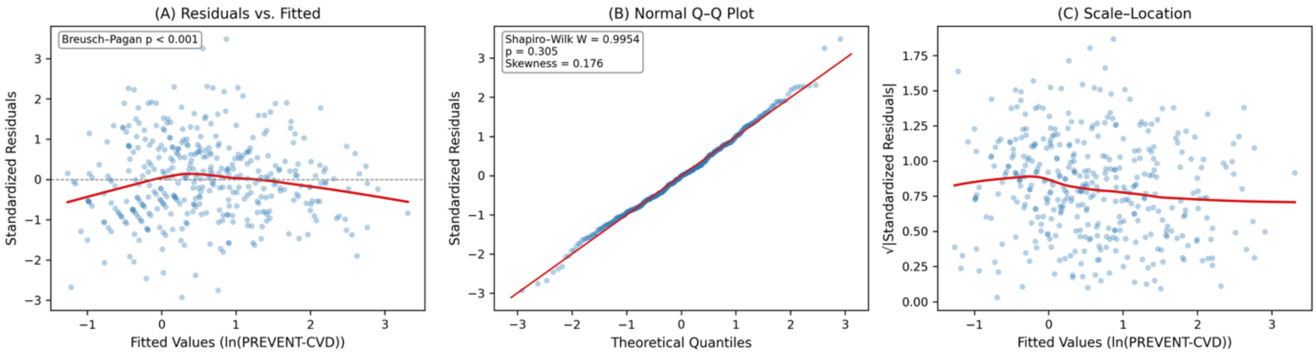

Supplementary Figure S2. Regression diagnostic plots for the full treadmill-augmented model (Model 1) with log-transformed PREVENT-CVD risk [ln(PREVENT-CVD)] as the dependent variable (n = 387). (A) Residuals vs. fitted values plot showing a substantially improved distribution with no systematic pattern and no negative predicted values. Residual heteroscedasticity remained statistically significant (Breusch–Pagan p <0.001) but was substantially attenuated compared with the untransformed model; heteroscedasticity-consistent (HC3) standard errors were used to ensure robust inference. (B) Normal Q–Q plot demonstrating approximate normality (Shapiro–Wilk W = 0.995, p = 0.305; residual skewness = 0.18). (C) Scale–location plot showing a more uniform spread of residuals across fitted values. Together, these diagnostics support the adequacy of log-transformation for the primary regression analyses.
